# Coherent soft X-ray diffraction imaging of coliphage PR772 at the Linac coherent light source

**DOI:** 10.1038/sdata.2017.79

**Published:** 2017-06-27

**Authors:** Hemanth K.N. Reddy, Chun Hong Yoon, Andrew Aquila, Salah Awel, Kartik Ayyer, Anton Barty, Peter Berntsen, Johan Bielecki, Sergey Bobkov, Maximilian Bucher, Gabriella A. Carini, Sebastian Carron, Henry Chapman, Benedikt Daurer, Hasan DeMirci, Tomas Ekeberg, Petra Fromme, Janos Hajdu, Max Felix Hanke, Philip Hart, Brenda G. Hogue, Ahmad Hosseinizadeh, Yoonhee Kim, Richard A. Kirian, Ruslan P. Kurta, Daniel S.D. Larsson, N. Duane Loh, Filipe R.N.C. Maia, Adrian P. Mancuso, Kerstin Mühlig, Anna Munke, Daewoong Nam, Carl Nettelblad, Abbas Ourmazd, Max Rose, Peter Schwander, Marvin Seibert, Jonas A. Sellberg, Changyong Song, John C.H. Spence, Martin Svenda, Gijs Van der Schot, Ivan A. Vartanyants, Garth J. Williams, P. Lourdu Xavier

**Affiliations:** 1 Laboratory of Molecular Biophysics, Department of Cell and Molecular Biology, Uppsala University, Husargatan 3 (Box 596), Uppsala SE-75124, Sweden; 2 SLAC National Accelerator Laboratory, 2575 Sand Hill Road, Menlo Park, California 94025, USA; 3 Center for Free-Electron Laser Science, DESY, Notkestrasse 85, 22607 Hamburg, Germany; 4 Centre for Ultrafast Imaging, University of Hamburg, Luruper Chaussee 149, 22761 Hamburg, Germany; 5 Australian Research Council Centre of Excellence in Advanced Molecular Imaging, La Trobe Institute for Molecular Science, La Trobe University, Melbourne 3086, Australia; 6 National Research Centre ‘Kurchatov Institute’, Akademika Kurchatova pl. 1, 123182 Moscow, Russia; 7 Argonne National Laboratory, 9700 S. Cass Ave, Lemont, Illinois 60439, USA; 8 Institut für Optik und Atomare Physik, Technische Universität Berlin, Hardenbergstraße 36, 10623 Berlin, Germany; 9 Department of Physics, University of Hamburg, Luruper Chausee 149, 22761 Hamburg, Germany; 10 Stanford PULSE Institute, SLAC National Accelerator Laboratory, 2575 Sand Hill Road, Menlo Park, California 94025, USA; 11 Biodesign Center for Applied Structural Discovery, Biodesign Institute at Arizona State University, Tempe, Arizona 85287-5001, USA; 12 School of Molecular Sciences, Arizona State University, Tempe, Arizona 85287-1604, USA; 13 Institute of Physics AS CR, v.v.i., Na Slovance 2, 18221 Prague 8, Czech Republic; 14 Biodesign Center for Immunotherapy, Vaccines, and Virotherapy, Biodesign Institute at Arizona State University, Tempe, Arizona 85287, USA; 15 Arizona State University, School of Life Sciences (SOLS), Tempe, Arizona 85287-5401, USA; 16 Department of Physics, University of Wisconsin Milwaukee, KIRC, 3135 N. Maryland Ave, Milwaukee, Wisconsin 53211, USA; 17 Department of Physics, Pohang University of Science and Technology, Pohang 37673, Korea; 18 Department of Physics, Arizona State University, Tempe, Arizona 85287, USA; 19 European X-ray Free Electron Laser GmbH, Holzkoppel 4, 22869 Schenefeld, Germany; 20 Department of Physics, National University of Singapore Blk S12, 2 Science Drive 3, Singapore 117551; 21 Centre for Bio-imaging Sciences, National University of Singapore Blk S1A, 14 Science Drive 4, Singapore 117557; 22 Division of Scientific Computing, Department of Information Technology, Science for Life Laboratory, Uppsala University, Husargatan 3 (Box 337), Uppsala SE-75105, Sweden; 23 Deutsches Elektronen-Synchrotron DESY, Notkestraße 85, D-22607 Hamburg, Germany; 24 Biomedical and X-Ray Physics, Department of Applied Physics, AlbaNova University Center, KTH Royal Institute of Technology, Stockholm SE-106 91, Sweden; 25 National Research Nuclear University MEPhI (Moscow Engineering Physics Institute), Kashirskoe shosse 31, 115409 Moscow, Russia; 26 NSLS-II, Brookhaven National Laboratory, Upton, New York 11873, USA; 27 Max-Planck Institute for the Structure and Dynamics of Matter, 22607 Hamburg, Germany

**Keywords:** X-ray tomography, Single-molecule biophysics, Virus structures, Biological physics

## Abstract

Single-particle diffraction from X-ray Free Electron Lasers offers the potential for molecular structure determination without the need for crystallization. In an effort to further develop the technique, we present a dataset of coherent soft X-ray diffraction images of Coliphage PR772 virus, collected at the Atomic Molecular Optics (AMO) beamline with pnCCD detectors in the LAMP instrument at the Linac Coherent Light Source. The diameter of PR772 ranges from 65–70 nm, which is considerably smaller than the previously reported ~600 nm diameter Mimivirus. This reflects continued progress in XFEL-based single-particle imaging towards the single molecular imaging regime. The data set contains significantly more single particle hits than collected in previous experiments, enabling the development of improved statistical analysis, reconstruction algorithms, and quantitative metrics to determine resolution and self-consistency.

## Background & Summary

Theoretical studies predict X-ray Free Electron Lasers (XFELs) can potentially image biomolecules and hence determine their structures without crystallization^1^. To realize this in practice has proved a significant experimental challenge. The Single Particle Imaging (SPI) Initiative^2^ was formed as a collaborative effort to identify and solve the experimental challenges to achieving high-resolution imaging of single molecules with X-rays.

Coliphage PR772 is a virus of approximately 70 nm in diameter, which infects *Escherichia coli* (*E. coli*). It was selected as the sample for this experiment due to its high structural homogeneity, uniform size distribution, suitable particle concentration in solution, having a known structure, and the ability to be aerosolized by Gas Dynamic Virtual Nozzle (GDVN)^3^ for injection into the XFEL beam using an aerosol injector. Having a known structure of PR772 (unpublished) enables validation of any subsequent data analysis steps.

For the data presented here, Coliphage PR772 was aerosolized and delivered into the focus of the LAMP instrument at the Atomic Molecular Optics (AMO) beamline^4^ of the Linac Coherent Light Source (LCLS) X-ray laser^5,6^. The data include clear diffraction snapshots from single PR772 virus particles.

## Methods

### Sample preparation

Bacteriophage PR772 (ATCC BAA-769-B1) infects *E.coli* K12 J53-1 (ATCC BAA-769). PR772 was cultured on agar by the overlay method. Tryptic Soy Agar (Difco Tryptic Soy Agar) was prepared and poured into petri dishes to form a hard agar support layer. The overlay medium was soft agar prepared with Tryptic Soy Broth (Bacto Tryptic Soy Broth) along with 0.5% Agar-Agar (Microbiology, Merck). *E. coli* was cultured in Tryptic Soy Broth to reach an OD_600_ of 0.7. Soft agar was melted and cooled to 45 °C. *E. coli* and viral stock (10^8^ pfu ml^‒1^) were added to the soft agar in a volume ratio of 10:1 and mixed well. The mixture was immediately poured onto the hard agar in the petri dishes and incubated overnight at 37 °C.

**Figure 1:**
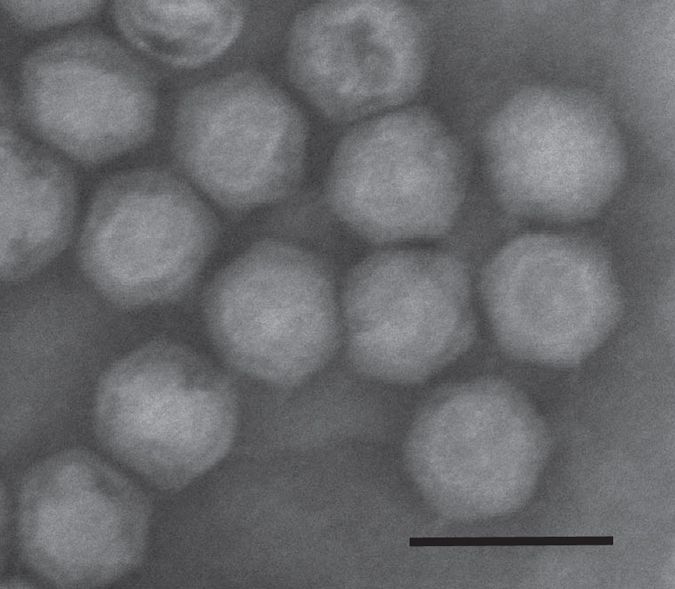
Electron micrograph of purified bacteriophage PR772. Scale bar represents 100 nm.

The soft agar layer from the overnight culture was scraped off and collected in a sterile container, diluted with 100 ml of sterile storage buffer (TRIS 50 mM, NaCl 100 mM, MgSO_4_ 1 mM, EDTA 1 mM, pH 8.0) and mixed overnight at 4 °C. The mixture was centrifuged at 8,000 g for 30 min to remove agar and cell debris. The supernatant was collected and filtered through a 0.45 micron filter (Filtropur S 0.45, Sarstedt). The viral particles were separated from the permeate solution by PEG precipitation (9% w/v PEG 8000 and 5.8% w/v NaCl). After mixing overnight at 4 °C, the precipitate was centrifuged for 90 min at 8,000 g. The supernatant was discarded and the viral pellet was suspended in 1 ml storage buffer. The viral suspension was then applied to a Capto-Q anion exchange column. The sample was eluted by varying the concentration of NaCl (100 mM–1.5 M). The fractions representing the elution profile peak was collected and observed with a electron microscopy (EM) (Quanta FEG 650, FEI) to confirm the presence of intact viral particles. (Figure 1)

Before sample injection, the PR772 was transferred from storage buffer into volatile ammonium acetate buffer (250 mM, pH 7.5) using PD10 desalting columns (GE Healthcare). Hence, most assays were performed with sample in ammonium acetate buffer instead of storage buffer.

### Sample characterization

#### Infectivity

Virus titers were measured by plaque assay following purification purification^7^. Serial 10-fold dilutions of purified virus were plated on a mat of *E. coli* and incubated overnight at 37 °C. Plaque forming units per ml (pfu ml^‒1^) were calculated using the formula: average # plaques/volume plated x dilution.

#### Liquid phase

**Figure 2:**
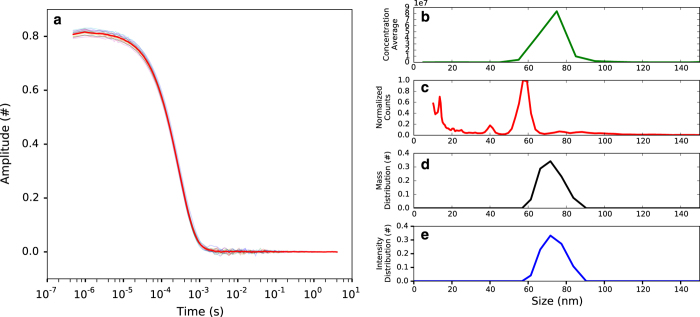
Sample characterization of PR772. NTA measurements (**b**) of bacteriophage PR772 in ammonium acetate buffer (250 mM, pH 7.5). The curve shows the distribution of the different sized particles in the solution. We observed a single large peak at 75 nm with concentration of 10^8^ particles/ml, which is similar to the dilution used for measurement. The diameter peak from the DMA measurement (**c**) was approximately 60 nm. The actual gas flow during the DMA measurement was lower than the set value, which resulted in a diameter low by about 10%. The gas flow was disregarded since the purpose of the DMA measurement was to assess sample purity and not size. DLS measurements of bacteriophage PR772 in ammonium acetate buffer (250 mM, pH 7.5). Curve in red shows the average of 18 correlation data collected over time for the sample (**a**). Curves in black and blue show the size of the particles based on mass (**d**) and intensity (**e**) distribution respectively. The mean diameter of the particle was 75.99 nm with a polydispersity index (PDI) of 0.012.

The size and monodispersity of the PR772 in ammonium acetate buffer were measured using Nanoparticle Tracking Analysis (NTA) (NanoSight LM10, Malvern Instruments Ltd.) (Figure 2) and Dynamic Light Scattering (DLS) (w130i, AvidNano Ltd.) (Figure 2). The sample was diluted to the required concentrations for measurement. For NTA 10^8^ particles per ml were used to limit the number of tracks to 200. For DLS about 10^10^ particles per ml was used to reach a recommended counts per s.

#### Gaseous phase

The size distribution in the gas phase was measured using Electrophoretic DMA (Figure 2). PR772 was aerosolized with a nano-electrospray ionization (ESI) source (TSI model 3480) and passed through an electrostatic classifier (TSI model 3480) to continuously classify particles from 10 to 500 nm. Classified particles were counted with a condensation particle counter (CPC, TSI model 3786).

#### Injection testing

Injection tests on PR772 were performed with a setup similar to the one subsequently used at the LCLS experiment to investigate the aerosolization characteristics of the samples and to study sample behavior during the injection procedure^8^. A glass microscope slide covered by a transparent sticky gel piece (GelPak) was positioned beneath the exit point of the aerodynamic lens (at a position similar to the interaction region with the X-ray beam at LCLS). Particle dusting spots were observed through an objective lens mounted below the glass slide. The focused particles from the injector were also collected on formvar/carbon grids (#01754F, F/C 400 mesh Cu, Ted Pella Inc.). These samples were examined by EM without any further modification.

### Sample injection at LCLS

Samples were delivered into the X-ray beam using the aerodynamic lens stack system used in previous experiments^9^. Purified PR772 was transferred to a volatile ammonium acetate buffer at a concentration of 10^12^ particles ml^−1^ and introduced to the injector via a GDVN^3^ at a flow rate of 1–2 μl min^−1^. The aerosol continued through a gas skimmer and a relaxation chamber, forming a fine beam of focused particles by the aerodynamic lens stack^10^. The focus of the particle beam and multiplicity of the particles could be optimized by regulating the flow of sample and gas along with the skimmer pressure.

### Experimental setup and data collection

Coherent diffraction snapshots using single soft X-ray pulses were recorded at the AMO beamline of the LCLS XFEL using the LAMP endstation^4^. The configuration was similar to that used in previous coherent diffraction experiments^11^, but with a shorter distance from the sample interaction region to the detector due to the gate valve between detector and sample chambers being removed for the present experiment.

Measurements were performed using LCLS tuned to a photon energy of 1.6 keV delivering 4 mJ into a 70 fs duration pulse at the end of the undulators. This was focused into a nominal 1.5 μm^2^ FWHM region using a pair of Kirkpatrick-Baez (KB) mirrors, giving a nominal power density of ~3.8 10^18^ W cm^‒2^ or 10^13^ photons per pulse assuming no beamline losses. Comparison of measured and calculated from known scattering objects indicate that the actual power density may differ from this estimate by a factor of 10. This is due to three combined effect of overfilling of the focusing optics, carbon contamination on the KB mirrors increasing mirror roughness (reducing reflectivity), and a less-than-perfect focal spot size due to carbon contamination distorting the KB mirrors (increasing effective focal spot size).

The photon energy was selected by considering the highest achievable resolution, while remaining below the silicon absorption edge at 1.8 keV. At photon energies above the silicon K-edge of 1.8 keV, the silicon beam conditioning apertures’ fluorescence increases the background. A series of silicon apertures, including a post-sample aperture, were used to limit the amount of background scatter incident on the detectors from the beamline. Three 1 mm×1 mm Si_3_N_4_ apertures were used to reduce low q scatter. The first two apertures were positioned laterally to the beam to form a small rectangle, with the third aperture used to clean up diffraction from the first two. The first two apertures were separated by 5 cm, with the third aperture separated by 10 cm. Additionally the third aperture was 10 cm away from the focus. A large round aluminum post sample aperture was used to block high q scatter. It was located 2.4 cm downstream of the sample with a diameter of 4 cm.

Diffraction snapshots were recorded using two pairs of pnCCD detectors^12^ at the LCLS pulse repetition rate of 120 Hz. One pair of pnCCD detectors was located 10 cm downstream of the interaction region, and a second pair of detectors further downstream at 58.1 cm from the interaction region. The pixel size are 75 μm and each detector in the pair has 512×1,024 pixels giving the full set-up a pixel count of two planes each with 1,024×1,024 pixels. The experimental configuration was similar to that described in ref. 13, albeit with a higher photon energy (1.6 keV instead of 1.2 keV), and the use of a different beam conditioning configuration and the front pnCCD. The majority of the data processing and evaluation of the quality of the data was conducted on the downstream detector. The resolution of the downstream detector is 11.6 nm at the edge or 8.3 nm in the corner giving an oversampling of the diffraction patterns of >10.

The data were analyzed online using *Hummingbird*^14^ and *psana*^15^.

### Data processing

Data saved in the native XTC format used at the LCLS were analyzed and converted to the CXI file format using the LCLS data analysis framework *psana*^15^. The raw pnCCD pixels contain analog to digital units (ADUs), to which various corrections must be made in order to obtain photon counts. As a first step, dark calibration and row-by-row common mode correction were performed on the pnCCD detector images by the LCLS software environment, *psana*. Data was calibrated using *psana*’s *ImgAlgos.NDArrCalib* module, with pedestal subtraction (*do_peds*), common-mode correction (do_cmod), statistical correction (do_stat) and gain corrections (do_gain) turned on. A flat-field data set using silicon K edge fluorescence (1.7 keV) was used to calibrate the gain on a pixel basis. These gain-corrected ADU values were then thresholded based on an average of 128 ADUs/photon in order to obtain photon counts.

Not all detector frames (or events) contained diffraction from single particles. Since the average number of particles per X-ray focus volume is much less than one, most of the pulses do not hit any particles. Hits (events in which a particle was intercepted by the X-ray beam) were identified using a chi squared metric adapted from ref. 16.

$$\chi_{j}^{2}=\frac{\overset{¯}{{\left(I_{j}-B_{j}\right)}^{2}}}{Var\left(B_{j}\right)}$$

Chi squared for the jth image *I*_*j*_ was calculated by subtracting a running median background *B* and normalizing by the variance of *B*. The chi squared value was calculated within an annular area of radii 150 and 400 pixels from beam center and hits were defined to frames with Chi-squared above 10. Hits were saved to file with detector corrections applied (i.e., pedestal, common mode and gain corrected) and then down-sampled by a factor of 4.

## Data Records

The data are deposited in the Coherent X-ray Imaging Data Bank (CXIDB)^20^ in the CXIDB data format, which is based on the HDF5 format (Data Citation 1). Convenient functions for accessing the CXIDB data file exist in the libspimage package for C and Python (Maia 2010), as well as many computing environments, including Python using the *h5py* module and MATLAB using e.g., the *h5read* function. The Owl software is convenient for visualizing data in the CXIDB format. In addition to the CXI file, the conversion script (create_dataset.py) and additional metadata files (selection.h5, psana.cfg) are provided along with usage instructions. Detector panel calibration files mapping data to real space are also provided. Configuration files for *Hummingbird* and *psana* are provided for completeness of describing processing performed on the deposited data.

## Technical Validation

**Figure 3:**
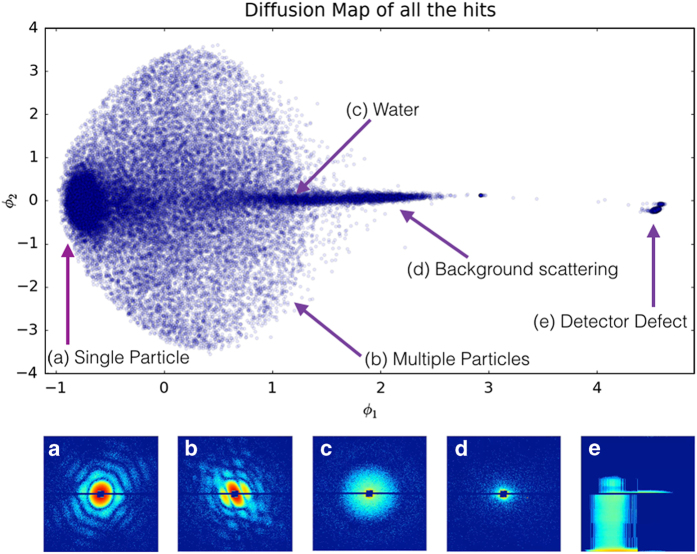
Diffusion map analysis of all the hits reduced to two dominant dimensions Φ_1_ and Φ_2_ as described in the text. Clustering helps identify the single particle diffraction patterns in the dataset. In addition to single hits (**a**) and multiple hits (**b**), the spherical particles (**c**) classified as ‘water’ may also contain contaminant residue from the sample and buffer. Additionally slightly larger than average background scattering (**d**) and frames with a defective readout (**e**) are also shown in the diffusion map.

**Figure 4:**
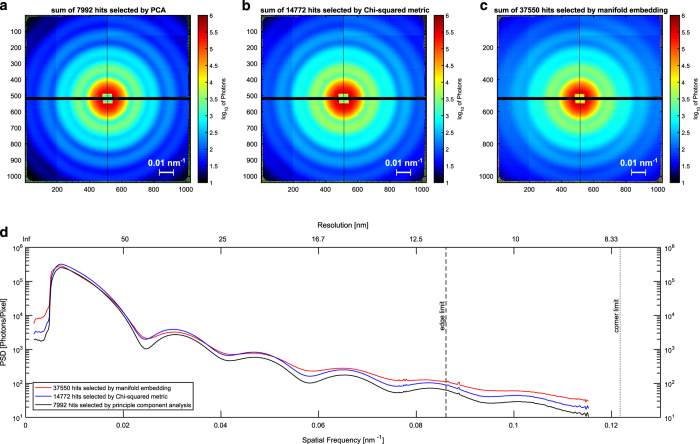
Comparison of single hit selections by principal component analysis (PCA), Chi-sqaured metric and manifold embedding. Summed diffraction patterns of (**a**) PCA, (**b**) Chi-squared metric and (**c**) manifold embedding. (**d**) Averaged signal over angle (power spectral density, PSD) as a function of spatial frequency and resolution. Five diffraction maxima are fully captured within the detector edges. The sixth maximum is captured within the detector corners.

Single-particle diffraction patterns were identified by analyzing all the hits in a reduced set of dimensions using diffusion map embedding similar to the method described in ref. 17 shown in Figure 3. The normalized graph Laplacian is used to calculate the likelihood of diffusion from the center of the single particle cluster at Φ_1_, Φ_2_, Φ_3_=−0.75, 0, 0. A likelihood value of 0.725 or higher is considered inside the single particle cluster. A total of 12,678 out of 14,772 images were identified as single particle hits. An alternative manifold-based data analytical approach yielded a larger dataset consisting of 37,550 single-particle snapshots, whose indices are provided. This approach reveals and corrects intensity variations and a range of other imaging artifacts^18^. Another method for data sorting and classification is based on principal component analysis (PCA)^19^. With this approach we have filtered 21,733 images as hits by intensity thresholding (all images above 200k photons). A total of 7,992 images were identified by the PCA technique as single hits and removed outliers by radial intensity filtering (also provided). A comparison of the summation of the single-particle hits, forming a pseudo small angle X-ray scattering patterns, is shown in Figure 4 and is shown in Table 1 per XTC run number.

Manual selection was not employed due to the large number of hits and the potential for user bias. Because identification of single-particle hits is dependent on the input parameters for clustering, we have deposited all data frames and identified potential single particle hits as a list of events. This enables the testing and comparison of different hit sorting algorithms.

Raw XTC files are included in the data deposition for anyone wishing to repeat the analysis from scratch.

## Additional Information

**How to cite this article:** Reddy, H. K. N. *et al*. Coherent soft X-ray diffraction imaging of coliphage PR772 at the Linac coherent light source. *Sci. Data* 4:170079 doi: 10.1038/sdata.2017.79 (2017).

**Publisher’s note:** Springer Nature remains neutral with regard to jurisdictional claims in published maps and institutional affiliations.

## Supplementary Material



## Figures and Tables

**Table 1:** Single particle hits data table.

**Run Number**	**Number of frames in XTC file**	**Number of hits by diffusion map embedding**	**Number of hits by principal component analysis**	**Number of hits by manifold embedding**
182	97,733	15	2	0
183	169,227	3	0	0
184	136,624	1	0	0
185	77,079	7	4	0
186	453,285	2,048	1,121	4,759
188	322,097	1,171	599	2,885
190	265,585	1,498	838	4,175
191	293,284	1,718	944	4,427
192	224,583	1,279	656	3,209
193	329,049	2,525	1,370	6,781
194	214,891	1,661	897	4,555
196	204,236	1,436	818	3,792
197	188,895	1,410	743	2,967
Total	2,976,568	14,772	7,992	37,550
This table describes the estimated single particle PR772 hits, per raw XTC file, using three different classification methods. Additionally it provided the number of data frames per run, raw XTC file, deposited in the CXIDB.				
